# Immune dysregulation in gestational diabetes mellitus: placental downregulation of CXCL9 and IL1RL1 and altered immune cell infiltration

**DOI:** 10.3389/fcell.2026.1803128

**Published:** 2026-05-29

**Authors:** Zhimei Zhou, Li Huang, Liying Sheng, Weihong Chen, Congmei Yang, Yajing Xie, Yueli Wang, Binbin Chen, Yumin Ke, Zhuna Wu

**Affiliations:** Department of Gynecology and Obstetrics, The Second Affiliated Hospital of Fujian Medical University, Quanzhou, Fujian, China

**Keywords:** CXCL9, gestational diabetes mellitus (GDM), IL1RL1, immune cell deconvolution, immunomodulation, placental transcriptomics

## Abstract

**Background:**

Immune dysregulation is implicated in pregnancy complications, particularly gestational diabetes mellitus (GDM). This study aimed to identify immune-related genes and characterize immune cell infiltration in GDM placentas.

**Methods:**

mRNA transcript expression profiles were retrieved from the GSE70493 dataset and integrated with immune-related genes (IRGs) extracted from the ImmPort database. Differentially expressed immune-related genes (DIRGs) were identified, followed by functional enrichment, protein-protein interaction (PPI) network, and immune infiltration analysis using CIBERSORT. Expression of candidate genes was validated by qRT-PCR and immunohistochemistry (IHC) in an independent cohort (50 GDM, 50 Non-GDM). Effect sizes (Cohen’s d) were calculated.

**Results:**

Eleven DIRGs were identified between GDM pregnancies and normal pregnancies without maternal complications. GO analysis revealed significant enrichment in processes such as adaptive immune response, humoral immune response, immunoglobulin complex, and antigen binding. KEGG pathway analysis indicated enrichment in the Th1 and Th2 cell differentiation and cytokine−cytokine receptor interaction. In the discovery dataset (after correction for technical replicates: n = 30 GDM, n = 25 Non-GDM), CXCL9 was significantly downregulated in GDM (log_2_FC = −0.62; Cohen’s d = −0.718, 95% CI: –1.27 to −0.166; p = 0.021), while IL1RL1 showed no significant difference (p = 0.053). In the independent validation cohort, both CXCL9 and IL1RL1 were significantly downregulated by qRT-PCR and IHC (p < 0.05). GDM placentas showed increased monocytes and macrophages M1, and decreased resting NK cells and T cells (p < 0.05).

**Conclusion:**

CXCL9 and IL1RL1 are associated with immune dysregulation in GDM, as supported by external validation. However, the discovery dataset did not support their use as diagnostic biomarkers. Further large-scale studies are needed to clarify their roles.

## Introduction

GDM is defined as diabetes diagnosed in the second or third trimester of pregnancy that is not clearly overt diabetes before gestation (ElSayed et al., 2024). According to the criteria established by the International Association of Diabetes and Pregnancy Study Groups (IADPSG), the global prevalence of GDM is estimated at 14.0% (Wang et al., 2022). Systematic reviews indicate that the incidence in mainland China is particularly high, reaching 14.8% (95% confidence interval [CI]: 12.8%–16.7%), suggesting it may bear the world’s largest burden of GDM cases (Gao et al., 2018). GDM significantly increases risks to maternal, fetal, and neonatal health. It is associated with an increased risk of preeclampsia, gestational hypertension, labor induction, cesarean delivery, postpartum depression, congenital malformations, large-for-gestational-age (LGA) infants, shoulder dystocia, preterm birth, and neonatal intensive care unit (NICU) admission (Ye et al., 2022; Hannah et al., 2021). The economic burden is substantial; in the United States alone, the direct costs attributable to GDM and its complications (including hypertension, preterm delivery, and neonatal metabolic and respiratory issues) are estimated at $1.6 billion annually (Sweeting et al., 2024). Furthermore, the consequences of GDM extend well beyond the perinatal period. Both mothers with a history of GDM and their offspring face elevated lifetime risks of developing type 2 diabetes, obesity, and cardiovascular disease (Lizárraga et al., 2023).

Beyond impaired insulin resistance (IR) and glucose intolerance, GDM is characterized by low-grade systemic and placental inflammation (Plows et al., 2018). Emerging evidence links immune dysfunction to GDM, suggesting that alterations in immune cell populations and function may contribute to adverse pregnancy outcomes (McElwain et al., 2021; Sharma et al., 2022). Supporting this, Lobo et al. (2018) documented a marked decrease in regulatory T cells (Tregs) accompanied by a rise in cytotoxic natural killer (NK) cells (CD56dimCD16+) in the peripheral blood of GDM patients. Furthermore, women with GDM exhibit a higher proportion of circulating proinflammatory CD4^+^ T cells and elevated proinflammatory-to-anti-inflammatory T cell ratios (Th17:Treg and Th1:Treg) compared to non-GDM during pregnancy (Sheu et al., 2018). This inflammatory state is reflected at the cytokine level. Multiple pro-inflammatory cytokines, including TNF-α, IL-1β, IL-6, IL-7, IL-8, and IL-15, are upregulated in GDM placentas and cord blood (Corrêa-Silva et al., 2018; Keckstein et al., 2020; Gomez Ribot et al., 2020; Li Y-x et al., 2020). Notably, increased inflammatory cell infiltration, macrophage numbers, and CCL2 expression in GDM are uncovered via placental analysis. It is worth noting that HFD-induced placental inflammation and GDM phenotypes in mice were ameliorated through the blocking of CCL2/CCR2 signaling – a key pathway for macrophage recruitment (Huang et al., 2023). Stimulation with IL-1β or lipopolysaccharide (LPS) is shown by *in vitro* studies to enhance the production of pro-IL-6, TNF-α, and IL-1β, along with increased expression of components in their respective signaling pathways (Zgutka et al., 2024).

While the precise mechanisms initiating the immune response in GDM remain incompletely understood, this study employed bioinformatics approaches to investigate DIRGs and elucidate the role of immunity in GDM pathogenesis. Recognizing specific immune-related genes as diagnostic biomarkers holds considerable potential for enhancing GDM diagnosis and management. Moreover, the relationship between immune cell profiles and GDM was described in our research. Therefore, the central objective of this study was to establish a highly efficient and specific diagnostic model based on immune-related gene signatures. This model aims to enhance the clinical diagnosis and discrimination of GDM, ultimately striving to improve patient prognosis and treatment outcomes.

## Methods

### Collection of data

The corresponding GDM dataset GSE70493 was retrieved from the GEO database (https://www.ncbi.nlm.nih.gov/geo/). GPL17586, the data platform of this dataset, is found to contain mRNA extracted from the placentas (maternal side) of 30 clinically confirmed GDM cases. In the raw data, one GDM sample and three non-GDM samples were each run in triplicate as technical replicates. Technical replicates were averaged to yield one expression profile per biological sample before differential expression analysis. Consequently, the final sample sizes used in all analyses were n = 30 for the GDM group and n = 25 for the non-GDM group. Data related to IRGs were sourced from the ImmPort database (Supplementary Table S1).

### Data processing

Technical replicates from the same biological sample were identified using a custom mapping file and averaged to obtain a single expression profile per biological sample before differential expression analysis (final sample sizes: n = 30 for GDM, n = 25 for non-GDM). Microarray probes were mapped to gene symbols according to the manufacturer’s annotation file. For genes detected by multiple probes, expression levels were represented by the mean value across probes. Normalization and background correction were performed using the ‘limma’ R package. Differentially expressed genes (DEGs) between GDM and Non-GDM placentas were identified with a threshold of |log_2_ fold change (FC)| ≥ 0.3 and Benjamini–Hochberg adjusted p-value (q-value) < 0.05. The same false discovery rate (FDR) correction was applied to all downstream analyses involving gene lists. Subsequently, the intersection of immune-related genes (IRGs) from the ImmPort database and DEGs was determined to identify differentially expressed immune-related genes (DIRGs).

Sensitivity analysis showed that the number of DEGs decreased sharply from 24 at |log_2_FC| ≥ 0.3 to 5 at |log_2_FC| ≥ 0.5, and further to zero at thresholds ≥0.8. Downregulated genes consistently outnumbered upregulated genes across all thresholds (Supplementary Figure S1). These findings suggest that most DEGs identified at the nominal threshold have modest effect sizes, while a small set of downregulated genes (e.g., those significant at |log_2_FC| ≥ 0.5) represent more robust signals. However, the absence of genes at higher thresholds (e.g., ≥0.8) may also reflect insufficient statistical power due to the limited sample size (n=30 GDM, n=25 Non-GDM) or biological heterogeneity within the GDM group, rather than an exclusive biological characteristic of placental tissue.

### Enrichment analyses of GO and KEGG pathways for DIRGs

We utilized the “DOSE,” “org.Hs.eg.db,” “clusterProfiler,” and “enrich plot” packages to carry out functional annotation analysis via GO and KEGG pathway enrichment. Within three GO categories, specifically biological processes (BP), cellular components (CC), and molecular functions (MF), as well as among curated KEGG signaling pathways, we systematically characterized the enriched terms. The “ggplot2” package, with its modular visualization framework, was used to generate dot plots for visualizing the enrichment results. We determined statistical significance for functional enrichment with a p-value threshold set at < 0.05.

### PPI and hub genes analysis

The STRING website (https://string-db.org/) was employed to look for a PPI network. In the “multiple proteins” module, 11 DIRGs were input, and in the organism module, “*Homo sapiens*” was selected. Researchers converted Protein IDs into gene symbols and removed PPIs lacking associated gene names. A confidence score threshold of 0.300 (medium confidence) was applied to retain interactions. Following that, Cytoscape 3.10.0 was used to develop the PPI network, with the cytoHubba plugin in Cytoscape employed to pinpoint hub genes.

### Selection of GDM prediction genes using DIRGs

LASSO and mSVM-RFE facilitated the identification of these biomarkers. LASSO regression was performed using the “glmnet” R package with 10-fold cross-validation to select the optimal penalty parameter λ (chosen as λ.min). Meanwhile, researchers employed the “e1071” R package to perform the mSVM-RFE algorithm. The mSVM-RFE algorithm was implemented using 10-fold cross-validation to rank features and determine the optimal number of predictive genes based on cross-validation accuracy. This algorithm, utilizing resampling methods in each iteration, aims to stabilize feature rankings and determine the most relevant features by removing feature vectors generated by the SVM through supervised machine learning techniques.

### Pregnant women and placental tissues

Fifty paraffin-embedded specimens from pregnant women with GDM and 50 from pregnancies without maternal complications, who were diagnosed at the Second Affiliated Hospital of Fujian Medical University (Fujian, China) between January 2021 and December 2024, were collected. All GDM patients were managed with lifestyle modifications (dietary counseling and exercise) alone throughout pregnancy, and none received insulin or oral hypoglycemic agents prior to delivery. The commencement of this study was approved by the Research Ethics Committee of the Second Affiliated Hospital of Fujian Medical University before data collection.

GDM was diagnosed according to the International Association of Diabetes and Pregnancy Study Groups (IADPSG) criteria using a 75-g oral glucose tolerance test at 24–28 weeks of gestation (fasting glucose ≥5.1 mmol/L, 1 h glucose ≥10.0 mmol/L, or 2 h glucose ≥8.5 mmol/L). Clinical and demographic characteristics of the validation cohort (50 GDM patients and 50 Non-GDM) are summarized in Table 1. All participants met the following inclusion criteria: singleton pregnancy, delivery at ≥37 weeks of gestation, and absence of preeclampsia, pregestational diabetes, chronic hypertension, or multiple gestation. The two groups were comparable in maternal age, gestational age at delivery, parity, birth weight, or mode of delivery, while pre-pregnancy BMI was significantly higher in the GDM group (p < 0.05), consistent with the known GDM phenotype.

**TABLE 1:** Baseline clinical and demographic characteristics of the validation cohort (GDM vs. Non-GDM).

Characteristic	GDM group (n=50)	Non-GDM group (n=50)	P Value
Maternal age (years), mean ± SD	34.02 ± 4.048	32.34 ± 4.583	0.0549
Pre-pregnancy BMI (kg/m^2^), mean ± SD	23.09 ± 3.212	21.85 ± 2.701	0.0392*
Gestational age at GDM diagnosis (weeks), mean ± SD	25.24 ± 1.322	–	–
Gestational age at delivery (weeks), mean ± SD	38.63 ± 1.091	38.93 ± 1.080	0.1715
Parity, n (%)	​	​	0.1050
– Primiparous	17 (34%)	25 (50%)	​
– Multiparous	33 (66%)	25 (50%)	​
Mode of delivery, n (%)	​	​	0.3149
– Vaginal	1 (2%)	0 (0%)	​
– Cesarean section	49 (98%)	50 (100%)	​
Birth weight (g), mean ± SD	3265 ± 518.2	3283 ± 373.4	0.8407
Exclusion criteria			
– Preeclampsia	0	0	–
– Pregestational diabetes	0	0	–
– Chronic hypertension	0	0	–
– Multiple gestation	0	0	–
– Intrahepatic cholestasis of pregnancy (ICP)	0	0	–

* GDM, diagnostic criteria: International Association of Diabetes and Pregnancy Study Groups (IADPSG) criteria using a 75-g oral glucose tolerance test at 24–28 weeks of gestation (fasting ≥5.1 mmol/L, 1-h ≥10.0 mmol/L, or 2-h ≥8.5 mmol/L).

*Data are presented as mean ± SD, or n (%). P values were calculated using an independent t-test (continuous variables) or chi-square test (categorical variables). *P < 0.05.

### Immunohistochemistry (IHC)

We used the IHC staining method with an anti-CXCL9 and an anti-IL1RL1 antibody to categorize the staining intensity ratio in specimens. The scoring criteria were as follows: In consideration of the proportion of positive cells among all tissue cells and the intensity of staining in positive cells, the experimental results were determined as follows: (A) Scoring according to the number of stained cells. When the number of positive cells was less than one-third of the total number of cells, it was assigned 1 point; when it ranged from one-third to two-thirds, 2 points were given; when it was greater than or equal to two-thirds, 3 points were scored (B) Scoring according to the staining intensity. If the staining was negative, 0 points were assigned; if it was light yellow, 1 point was given; if it was brownish yellow, 2 points were assigned; if it was tan, 3 points were scored. The total score was calculated as A multiplied by B. Subsequently, the slide samples were divided into low-expression and high-expression groups. The low-expression group was defined by a total score of less than 6, and the high-expression group by a total score of 6 or greater. All IHC slides were independently evaluated by two experienced obstetric pathologists who were blinded to the clinical diagnosis. Inter-observer agreement was assessed using Cohen’s kappa coefficient (https://www.graphpad.com/quickcalcs/kappa1/). The kappa values were 1.00 ± 0.00 (95% CI: 1.00–1.00) for CXCL9 and 0.90 ± 0.043 (95% CI: 0.815–0.985) for IL1RL1, indicating almost perfect agreement (Supplementary Table S2). Discrepancies were resolved by consensus.

### Quantitative real-time PCR (qRT-PCR)

qRT-PCR was performed on fresh placental tissue obtained from a subset of the validation cohort: 19 GDM and 19 Non-GDM pregnancies. Total RNA was extracted using Trizol reagent (Beyotime, Biotechnology, China). cDNA synthesis was carried out according to the manufacturer’s instructions (TaKaRa, Japan). GAPDH was used as an internal reference gene, based on its documented expression stability in GDM placental tissues and its established use in prior studies of this experimental context (Li Y-x et al., 2020; Corrêa-Silva et al., 2018; Keckstein et al., 2020). Relative mRNA expression levels of CXCL9 and IL1RL1 were determined by the 2^−ΔΔCT^ method. Each sample was run in technical triplicate, and three independent experimental replicates were performed. The primer sequences utilized in this study are listed as follows:

CXCL9
  →: 5′- CTG​ATT​GGA​GTG​CAA​GGA​ACC-3′,
  ←: 5′- GGT​CTT​TCA​AGG​ATT​GTA​GGT​GG-3’.
IL1RL1
  →: 5′- TAA​TGT​GAT​GAC​TGA​GGA​CGC​A-3′,
  ←: 5′- GCT​CCG​ATT​ACT​GGA​AAC​AGA​G-3’.
GAPDH
  →: 5′-GTC​TCC​TCT​GAC​TTC​AAC​AGC​G-3′,
  ←: 5′-ACC​ACC​CTG​TTG​CTG​TAG​CCA​A-3′.

### Distribution of immune cells in GDM

The CIBERSORT algorithm served to quantify the proportion of 22 infiltrating immune cells in GDM, and we performed correlation analysis with the “Corrplot” package in R software. We used the “Vioplot” package to explore the 22 immune cells infiltration difference between the pregnancies without maternal complications and GDM.

### Methodological considerations for immune cell deconvolution in microarray data

We applied the CIBERSORT algorithm to microarray-derived gene expression data. CIBERSORT was originally developed and benchmarked using microarray data (Affymetrix HGU133A) with the LM22 signature matrix derived from microarray expression profiles. However, cross-platform applicability considerations remain relevant when applying CIBERSORT to different microarray platforms or when comparing with RNA-seq data. To assess the robustness of our findings and to mitigate potential platform-specific biases, we additionally employed MCP-counter — a method explicitly designed for immune cell quantification in transcriptomic data, including microarray platforms — as an independent validation approach.

### Statistical methods

R software (v.4.3.3) was utilized for all statistical analyses. We applied the Mann-Whitney U test to make comparisons between different groups and employed the Chi-square test for comparing 2x2 contingency tables. Analyses such as ROC analysis, SVM-RFE algorithm, LASSO regression, and Spearman correlation were carried out. Cohen’s d (Hedges-corrected) was computed to evaluate the effect size of CXCL9 and IL1RL1 expression differences between GDM and Non-GDM groups, using the effsize R package. Effect sizes are reported with 95% confidence intervals. A P-value of less than 0.05 was considered statistically significant.

## Result

### DIRG’s expression in GDM

**FIGURE 1:**
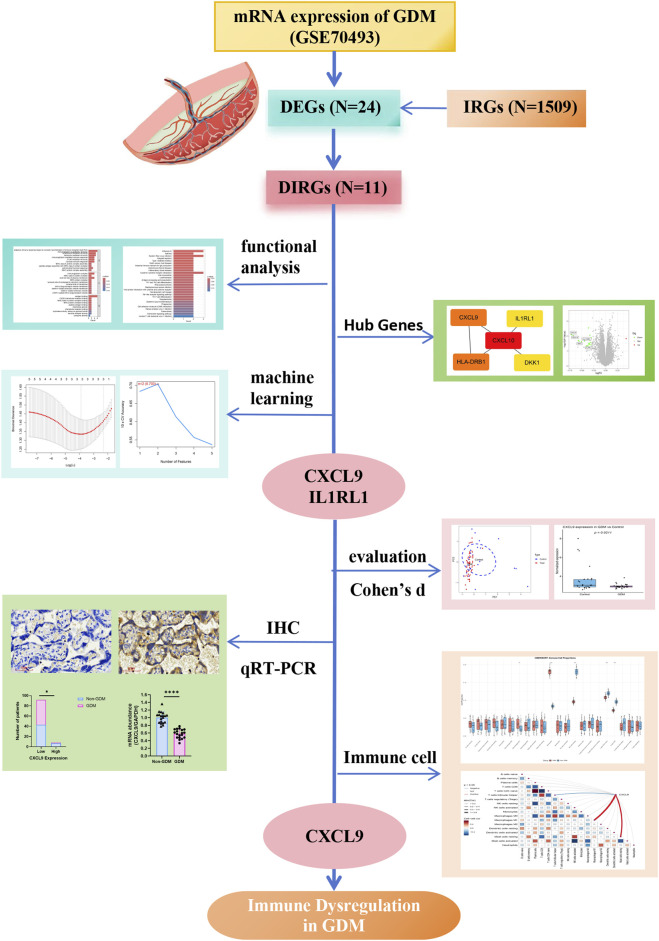
The flowchart of the overall study.

**FIGURE 2:**
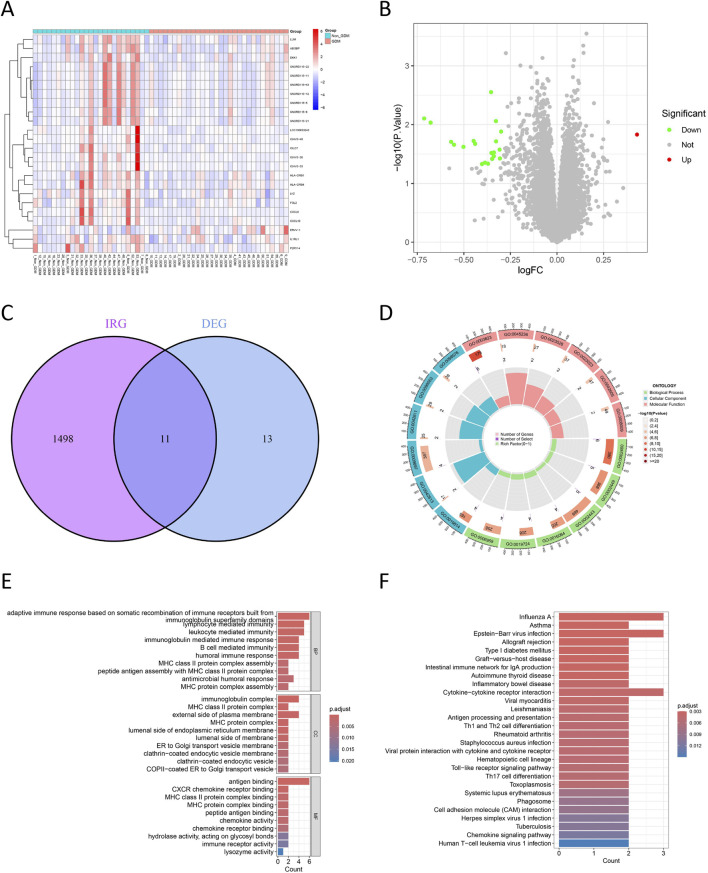
Identification and Function of DIRGs **(A)** Heatmap showing expression levels of the DEGs between GDM and non-GDM tissues from GEO. Genes are labeled on rows; sample IDs (columns) are omitted. Color gradient (red = high expression, blue = low) indicates relative expression **(B)** Volcano plot of 24 DEGs between GDM and non-GDM at nominal p < 0.05 (uncorrected). No genes met the FDR-corrected threshold (q < 0.05). Green dots: downregulated genes; black dots: non-differential genes **(C)** Venn diagram intersection identifies 11 DIRGs from DEGs and IRGs **(D)** GO analysis of 11 DIRGs (circle plot) **(E)** GO analysis of 11 DIRGs (bar graph) **(F)** KEGG pathway annotation of DIRGs (bar graph).

Figure 1 shows the flowchart related to the Overall analysis in GDM. At an FDR-corrected threshold of q < 0.05, no genes were significantly differentially expressed. Using a nominal p < 0.05 (uncorrected), we identified 24 downregulated DEGs between 30 GDM and 25 Non-GDM placentas (Figure 2A; Supplementary Table S3). Of note, all 24 DEGs were downregulated in GDM placentas, with no upregulated genes meeting the nominal threshold (Figure 2B). This unidirectional pattern may reflect the limited sample size (n=30 GDM, n=25 Non-GDM) and modest effect (see sensitivity analysis, Supplementary Figure S1) rather than a true biological absence of upregulated transcripts in GDM. Intersection with immune-related genes yielded 11 DIRGs (Figure 2C; Supplementary Table S4). All subsequent analyses involving these DIRGs are exploratory, and their biological relevance is supported by external validation (see below).

### GO and KEGG pathway enrichment analyses

To examine the potential biological functions and signaling pathways related to the 11 DIRGs, functional enrichment analysis was conducted using the “ClusterProfiler” package in R. The BP were mainly related to the humoral immune response, adaptive immune response, and immunoglobulin-mediated immune response. The CC of the DIRGs mainly centered on the immunoglobulin complex and the MHC class II protein complex. The MF of the DIRGs were significantly enriched in antigen binding and CXCR chemokine receptor binding (P < 0.05, Figures 2D,E, Supplementary Material S1). Moreover, KEGG enrichment findings demonstrated that the 11 DIRGs were mainly involved in Antigen processing and presentation, Th1 and Th2 cell differentiation, and Cytokine−cytokine receptor interaction. The above results collectively indicate GDM is closely related to immunity, which will promote further exploration of the potential mechanisms underlying GDM development (P < 0.05, Figure 2F, Supplementary Material S2).

### PPI network and hub gene selection

**FIGURE 3:**
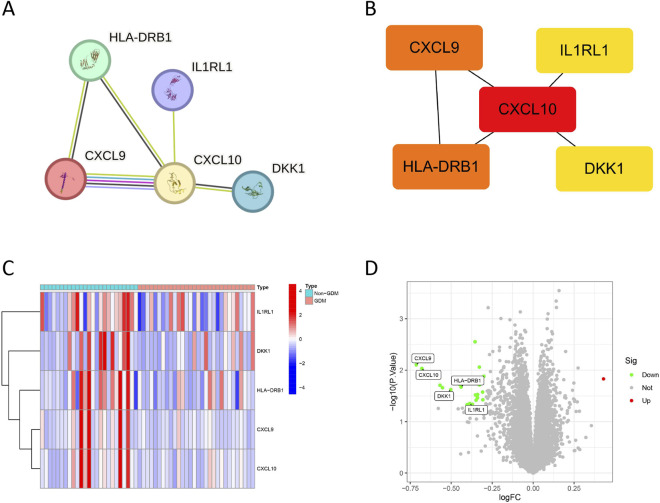
Association between DIRGs and Hub Genes **(A)** PPI network of five DIRGs and their interacting proteins, constructed using STRING **(B)** Hub genes identified via the MCC algorithm **(C)** Heatmap comparing expression of five hub genes in GDM vs. non-GDM placental tissues **(D)** Volcano plot of DEGs in GDM vs. non-GDM placental tissues, highlighting five hub genes.

A PPI network was constructed using the STRING database. The 11 DIRGs were input into the “multiple proteins” module, and *Homo sapiens* was selected as the target species. After the disconnected nodes were removed, 5 interconnected DIRGs were retained in the PPI network (Figure 3A). Of the 11 DIRGs, 6 (LYZ, IGHV3-30, IGLC7, IGHV3-48, IGHV3-33, and HLA-DRB4) did not form any retained interactions at the medium confidence threshold (STRING score ≥0.300), suggesting they may function as isolated nodes or interact with partners not captured by this network. This observation does not exclude their biological relevance, but indicates that the remaining 5 DIRGs (CXCL9, CXCL10, DKK1, IL1RL1, and HLA-DRB1) constitute a tightly interconnected subnetwork within the STRING database. Subsequently, the network genes were subjected to cluster analysis using the cytoHubba plugin in Cytoscape software. Five hub nodes were identified and categorized, which were prioritized by the MCC (Maximal Clique Centrality) algorithm (Figure 3B). The expression levels of IL1RL1, CXCL9, CXCL10, DKK1, and HLA-DRB1 genes were observed to be downregulated (Figures 3C,D).

### Relationship of prospective biomarkers in GDM

**FIGURE 4:**
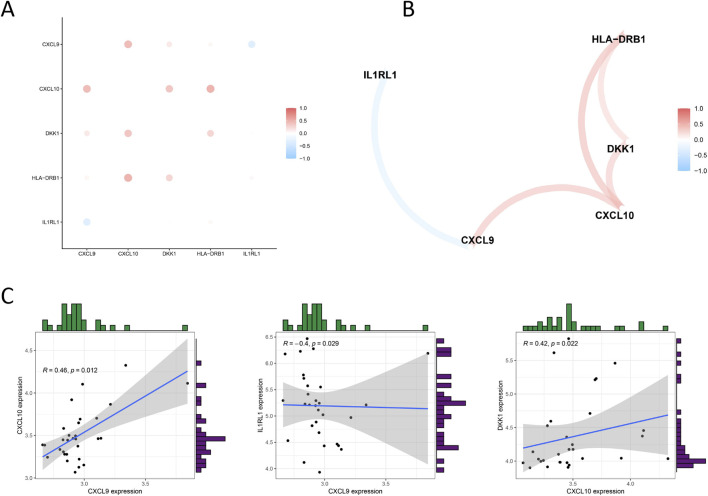
Correlation analysis among DIRGs **(A)** Correlation matrix of the five DIRGs. Color intensity denotes the Spearman correlation coefficient (r), with red indicating positive correlation and blue indicating negative correlation **(B)** Co-expression network of the five DIRGs retained in the protein–protein interaction network. Nodes represent individual genes; edges indicate significant pairwise correlations. Line thickness is proportional to the absolute value of the Spearman correlation coefficient **(C)** Scatter plots for selected DIRG pairs with high correlation coefficients. Each dot represents one placental sample. The shaded area represents the 95% confidence interval of the linear regression fit. Spearman correlation coefficients (r) and P-values are annotated in each panel. Statistical significance symbols: *P < 0.05, **P < 0.01, ***P < 0.001. Abbreviations: CXCL9, C-X-C motif chemokine ligand 9; CXCL10, C-X-C motif chemokine ligand 10; DKK1, Dickkopf-1; IL1RL1, interleukin-1 receptor-like 1.

Using a cutoff of 0.3, Spearman correlation analysis was performed to explore the interrelationships among the 5 DIRGs. With R’s ‘tidyverse’ and ‘corrr’ packages, we generated co-expression networks (Figure 4A) and correlation plots (Figure 4B) to visualize the results. Scatter plots indicated that there was a significant positive correlation between the expression level of CXCL10 and the levels of CXCL9 and DKK1. Moreover, the expression level of IL1RL1 was inversely correlated with CXCL9 expression (Figure 4C).

### Candidate genes for GDM

**FIGURE 5:**
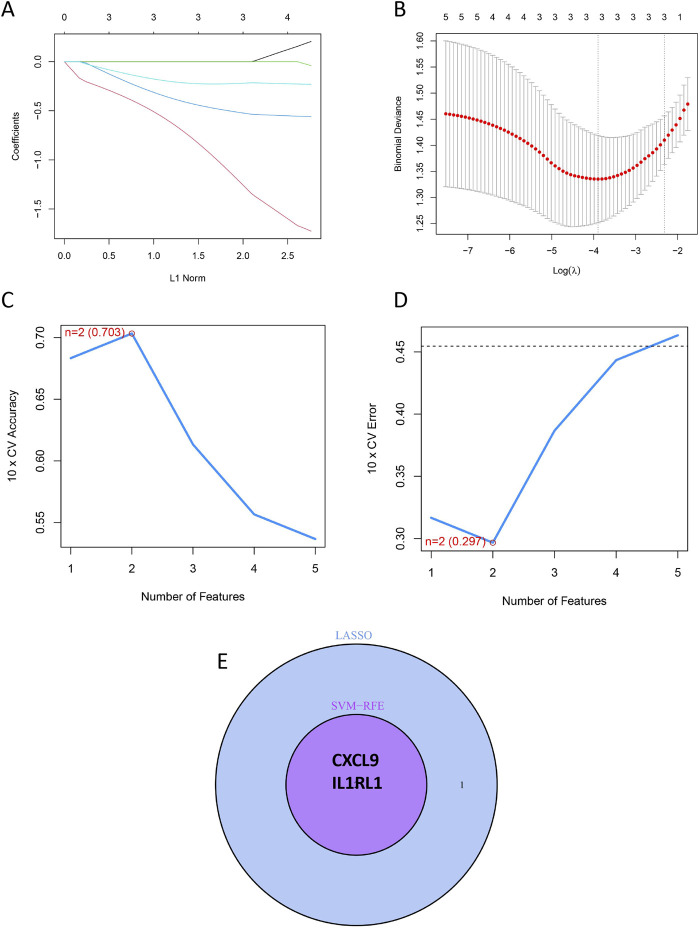
Machine learning-based feature selection for candidate genes in GDM **(A)** LASSO coefficient profiles of the DIRGs. Each colored line traces the coefficient value of an individual gene as the penalty parameter λ varies **(B)** Partial likelihood deviance as a function of log(λ) in LASSO regression. The left vertical dashed line indicates the optimal λ value (λ.min) that minimizes the deviance. The numbers along the top denote the number of genes with non-zero coefficients at each λ value **(C)** Ten-fold cross-validation accuracy of the SVM-RFE model as a function of the number of features retained. The highest accuracy (0.70) is achieved with 2 features **(D)** Ten-fold cross-validation error (misclassification rate) as a function of the number of features, with the lowest error (0.29) at 2 features. The optimal feature set size (k = 2) corresponds to CXCL9 and IL1RL1 **(E)** Venn diagram showing the overlap of candidate genes selected by LASSO regression and mSVM-RFE algorithms. Two genes (CXCL9 and IL1RL1) were consistently identified by both methods. Abbreviations: LASSO, Least Absolute Shrinkage and Selection Operator; mSVM-RFE, multiple Support Vector Machine-Recursive Feature Elimination; GDM, gestational diabetes mellitus.

To accurately identify candidate genes in GDM. Through the Lasso algorithms (Figures 5A,B), we selected 3 DIRGs (CXCL9, IL1RL1, and DKK1) as putative genes. In addition, the SVM-REF (Figures 5C,D) algorithms were also utilized to identify potential diagnostic biomarkers; two DIRGs (CXCL9 and IL1RL1) were selected as putative genes. Finally, the LASSO and mSVM-RFE were intersected to obtain 2 putative genes (CXCL9 and IL1RL1) for GDM, as presented in Figure 5E.

### Effect sizes and diagnostic performance of CXCL9 in the discovery dataset

**FIGURE 6:**
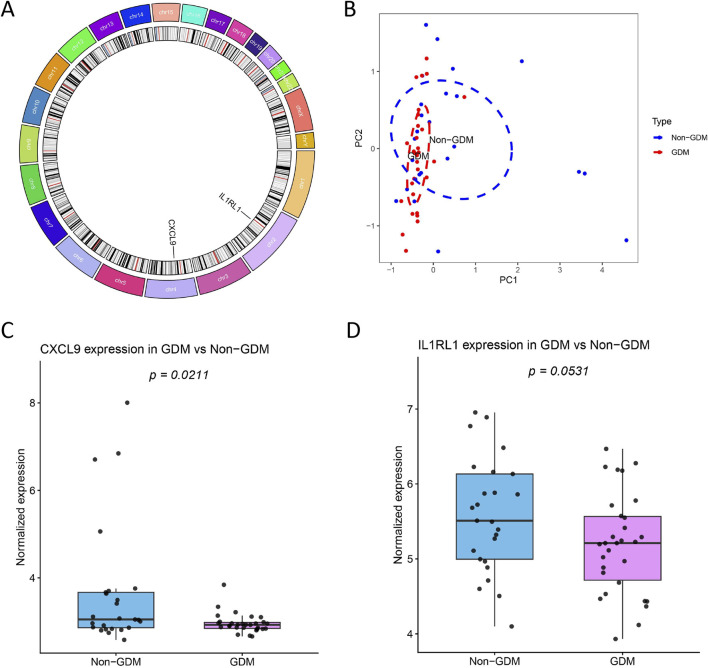
Additional analysis of two key DIRGs **(A)** The positions on the chromosome of the right crucial DIRGs **(B)** The PCA plot displays the sample distribution based on the expression profiles of 2 key DIRGs **(C)** The comparative expression levels of CXCL9 in GDM as opposed to non-GDM are presented by the GDM group datasets (P=0.0211) **(D)** The comparative expression levels of IL1RL1 in GDM as opposed to non-GDM are presented (P=0.0531).

The location of 2 DIRGs on the chromosome is presented in Figure 6A. Results of principal component analysis (PCA) indicated that the 2 putative genes had strong discriminatory ability between GDM and non-GDM, implying their crucial roles in GDM diagnosis (Figure 6B). Among the 11 DIRGs, CXCL9 was significantly downregulated in GDM placentas compared to Non-GDM (mean ± SD: 2.96 ± 0.22 vs. 3.67 ± 1.43; log_2_FC = −0.62; Cohen’s d = −0.718, 95% CI: 1.27 to −0.166; p = 0.021; Figure 6C). However, receiver operating characteristic (ROC) analysis showed a modest area under the curve (AUC) of 0.655 (95% CI: 0.497–0.812), with sensitivity 20% and specificity 40% at the optimal cut-off (Youden’s index = −0.4), indicating no clinically useful discriminatory ability (Supplementary Table S5). IL1RL1 was not significantly different between the two groups (p = 0.21; Cohen’s d = −0.536, 95% CI: 1.081 to 0.009; Figure 6D). Therefore, no diagnostic model or nomogram was constructed from this dataset.

### Independent validation using qRT-PCR and IHC

**FIGURE 7:**
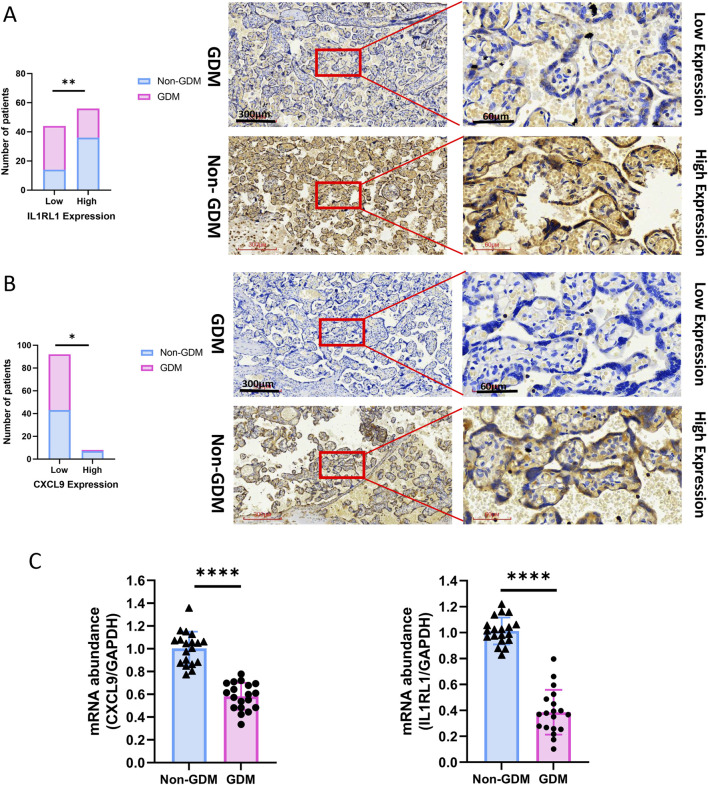
Independent validation using qRT-PCR and IHC **(A)** Significantly low expression of IL1RL1 was found in GDM **(B)** Significantly low expression of CXCL9 was found in GDM. Distribution of patients with 50 GDM and 50 normal pregnancies stratified by IL1RL1 and CXCL9 expression levels. The bar plot illustrates the number of patients with GDM and non-GDM categorized according to IL1RL1 and CXCL9 expression levels (Low vs. High; y-axis: Number of patients). Statistical analysis using the Chi-square test. *p < 0.05, **p < 0.01. Representative images (×40 and ×200) of IHC staining for CXCL9 and IL1RL1 in GDM and non-GDM specimens (high expression versus low expression) **(C)** qRT-PCR validation of CXCL9 and IL1RL1 expression in GDM vs. Non-GDM placentas. qRT-PCR was performed on fresh placental tissue from n = 19 GDM and n = 19 Non-GDM pregnancies. Each dot represents one biological sample. Bars show mean ± SD. ****p < 0.0001. P-values were calculated by an unpaired t-test.

In an independent cohort of 50 GDM and 50 Non-GDM placentas, IL1RL1 and CXCL9 expression in GDM and non-GDM were further assessed by IHC, and lower expression of IL1RL1 and CXCL9 was discovered to be markedly associated with GDM (Figures 7A,B, P_IL1RL1_=0.0013, P_CXCL9_ = 0.0270). We then performed qRT-PCR on fresh placental tissue from a subset of the same cohort (n = 19 GDM, n = 19 Non-GDM). Consistent with the IHC findings, CXCL9 and IL1RL1 mRNA levels were significantly downregulated in GDM compared to Non-GDM (Figure 7C; p < 0.05 for both). The discrepancy for IL1RL1 between the discovery dataset and the validation cohort is discussed below.

### Distribution of immune cells in GDM and the CXCL9 gene

**FIGURE 8:**
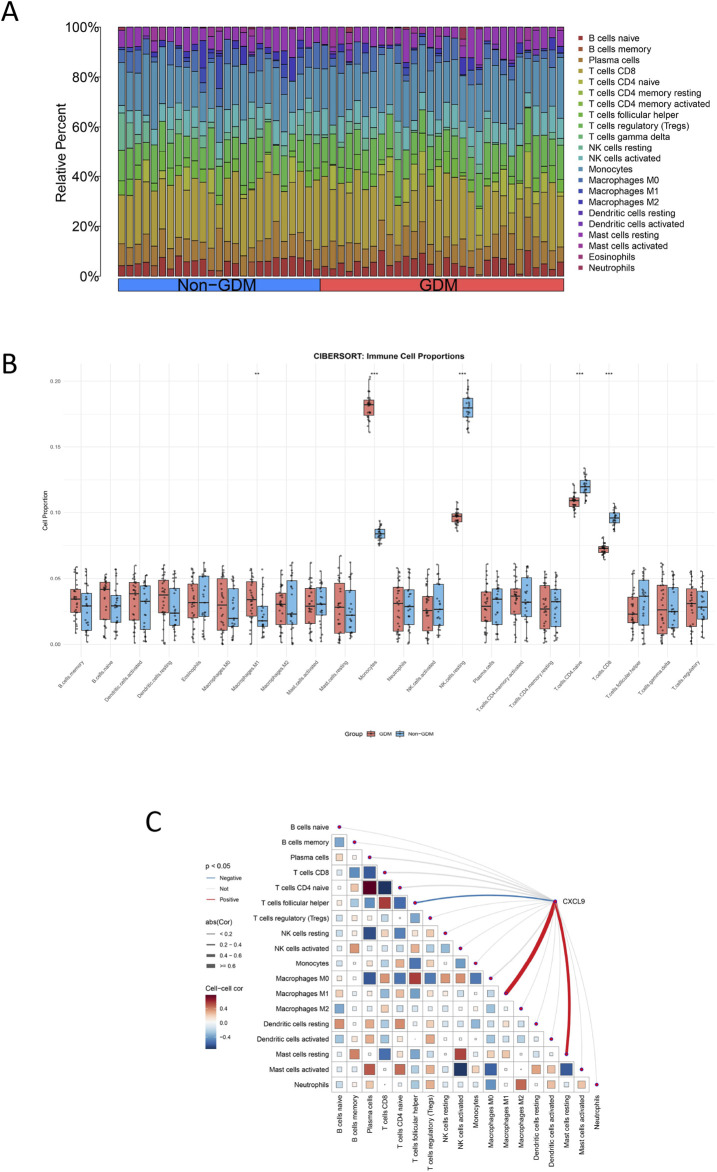
The evaluation of CXCL9 related to immune infiltration in GDM **(A)** Stacked bar plot showing the relative abundance of 22 immune cell subtype proportions between GDM and non-GDM samples **(B)** Differential immune cell infiltration profiles between GDM and non-GDM **(C)** Correlation between CXCL9 and infiltrating immune cells in GDM.

To gain a deeper insight into the relationship between GDM and immune cell infiltration, we used the CIBERSORT algorithm to compute the relative proportions of 22 immune cell types in both GDM samples and Non-GDM (Figure 8A; Supplementary Table S6). Subsequently, the immune cell infiltration in GDM samples and non-GDM samples was compared. It was revealed by the results that the abundances of Monocytes and Macrophages M1 were significantly higher in GDM, while the sparse infiltration of resting NK cells and T cells was observed in the GDM group (P < 0.05) (Figure 8B). These findings support the intimate connection between GDM and immune activity, underlining its potential importance in modulating immune cell function in GDM. Furthermore, we investigated the correlation between the CXCL9 gene associated with various infiltrating immune cells. The findings revealed that the CXCL9 expression exhibited a positive correlation with Macrophages M1 cells and resting mast cells, while showing a negative correlation with Follicular helper T cells (P < 0.05) (Figure 8C). These results underscore that CXCL9 is closely linked to immune activity and has significant implications for regulating immune cell dynamics in GDM.

### Validation of immune cell infiltration using MCP-counter

To evaluate the consistency of immune deconvolution results across methodologies, we performed an independent analysis using MCP-counter. Comparison of immune deconvolution results between CIBERSORT and MCP-counter revealed both concordant and discordant findings (Supplementary Table S7). For T lymphocytes, both methods indicated a reduction in GDM placentas (MCP-counter fold change = 0.987, p = 0.033; CIBERSORT fold change = 0.755, p < 0.001), supporting the robustness of this observation. However, for resting NK cells, the results were discordant: CIBERSORT showed a significant reduction (fold change = 0.537, p < 0.001), whereas MCP-counter showed no significant difference (fold change = 0.991, p = 0.145). This discrepancy may reflect differences in algorithm design, cell type definitions, or sensitivity to microarray-specific noise. The NK cell finding should therefore be interpreted cautiously.

## Discussion

GDM is among the most common endocrine disorders of pregnancy, manifesting primarily as hyperglycemia and glucose intolerance. The condition is characterized by an atypical immune response with low-grade inflammation, originating from dysregulation of the maternal immune system (Omazić et al., 2024). Components of both the innate and adaptive immune systems react to hyperglycemia and insulin resistance (IR), resulting in excessive immune cell infiltration into tissues, heightened cellular activation, and the release of elevated levels of inflammatory mediators into the maternal circulation (McElwain et al., 2021). Placental inflammation and hormonal imbalance play a central role in the pathophysiology of GDM(Calvo et al., 2024), with increased expression of tumor necrosis factor-α (TNF-α) and interleukin-6 (IL-6) in the GDM placenta exacerbating local inflammation and promoting insulin resistance (Golbidi and Laher, 2013). Despite these established links, the relationship between DEGs in GDM placental tissue and immune cell infiltration remains incompletely characterized. We therefore sought to identify immune-related DEGs associated with GDM and to define the composition and functional implications of infiltrating immune cells in the GDM placenta.

GO/KEGG enrichment analysis revealed that Toll-like receptor and chemokine signaling pathways were involved in the development of GDM. The PI3K/Akt and the MAPK pathways are the main insulin signaling pathways. The primary metabolic function of insulin is directed by the PI3K/Akt pathway, and the mitogenic effects of insulin are regulated by the MAPK(Ortiz-Huidobro et al., 2021). Increased release of proinflammatory cytokines induces insulin resistance in GDM(Jiménez-Escutia et al., 2023; Liu et al., 2024; Wang et al., 2023; Ma et al., 2022). Interaction with IL-1 receptor 1 (IL1R1) stimulates nuclear factor (NF)-κB pathways, leading to heightened production and release of other inflammatory mediators (e.g., TNF-α and IL-6) and thus initiating a self-amplifying autocrine cytokine network (Maedler et al., 2006). Insulin signaling inhibition is primarily mediated by increased serine phosphorylation of insulin receptor substrate 1 (IRS-1) (Heerwagen et al., 2010). Inflammation and insulin resistance can be triggered by TNF-α through enhancing pro-inflammatory cytokines and serine phosphorylation of IRS-1 via the MAPK and NF-κB pathways (Park et al., 2022), and reducing the tyrosine kinase activity of the insulin receptor (Barbour et al., 2007). c-Jun-NH2-terminal kinase (JNK)-dependent serine phosphorylation of IRS1 is elevated by IL-1β, thereby suppressing insulin sensitivity (Mohan et al., 2023). Thereafter, insulin-PI3K/Akt signaling in insulin-targeted tissues and cells is regulated by increased p-IRS1 (Robbins Gregory et al., 2014). Toll-like receptor (TLR) family members play a key role in the development of chronic inflammation and insulin resistance (Li B. et al., 2020). TLR-dependent inflammatory pathways are stimulated by hyperglycaemia, as shown by *in vitro* studies, with macrophages producing excessive pro-inflammatory cytokines as a result (Sisino et al., 2013; Wen et al., 2006). TLR4 can activate NF-κB and induce an inflammatory response through MyD88-dependent (Sindhu et al., 2016; Ahmad et al., 2018) and MyD88-independent pathways (Huang et al., 2012). Zgutka et al. (Zgutka et al., 2024) noted there was significant upregulation of IL1R1, IL1 receptor accessory protein (IL1RAP), TLR4, nuclear factor NF-kappa-B p65 subunit (RELA), and CD14 in placental samples obtained from GDM women. Then, the key involvement of the IL-1β pathway and TLRs in GDM pathogenesis was confirmed by the application of selected inhibitors of NF-κB, MAPK, and recombinant interleukin 1 receptor antagonist (IL1RA). The release of syncytiotrophoblast debris is antagonized by the interaction between chemokine concentration and the maternal immune microenvironment, a process that leads to systemic inflammation and placental oxidative stress (Lekva et al., 2016). Impaired angiogenesis, trophoblast dysfunction, and maternal-fetal immune tolerance are linked to chemokine imbalance (Zhang et al., 2022). Levels of some chemokines were found to be higher in GDM patients than in non-GDM patients, and levels of other chemokines (CCL4, CCL11, and CXCL10) were found to be lower in GDM, according to the meta-analysis and systematic review (Liu et al., 2022). Inhibiting the activation of the TLR4/NF-κB signaling pathway reduced the secretion of pro-inflammatory cytokines and chemokines, alleviating systemic inflammation and IR in GDM mice (Liu et al., 2024). These evidences indicated that IRG can affect inflammation and IR by modulating chemokine and Toll-like receptor signal pathway.

Based on three IRGs with the largest differences between the two groups, two genes (CXCL9 and IL1RL1) were filtered by the LASSO and mSVM-RFE. A low-molecular-weight protein secreted by interferon-γ stimulation is CXCL9, induced by Interferon-γ. They are produced by different cell types, encompassing immune cells like dendritic cells or macrophages and non-immune cells (Wightman et al., 2015; Amatschek et al., 2011). CXC chemokine receptor 3 (CXCR3) is bound and activated by CXCL9, with primary expression on the surface of immune cells (Müller et al., 2010). The receptor CXCR3 is activated by it through its N-terminal interaction with the receptor’s N-terminal and extracellular structural domains. Chemotaxis is induced by CXCL9, which participates in immune and inflammatory processes through directing T cells and NK cells to migrate to inflammation sites. An important role in tumor immunosurveillance and antitumor immunity is played by CXCL9 (Fukuda et al., 2020; House et al., 2020). The differentiation of Treg and Th17 cells can be affected by CXCL9 via the JNK pathway (Li et al., 2021). An animal experiment showed that both CXCL9 and CXCL10 played a critical role in modulating the infiltration and migration of basophils and NK cells into the immune microenvironment of islets, a process that impaired insulin secretion and function in mice with CXCR3 gene deletion (Burke et al., 2016). A bioinformatics study found that CXCL9/10 is significantly enriched in the TLR signaling pathway, suggesting a key role in GDM pathogenesis is played by it through regulating the inflammatory pathway via the signaling pathway (Wang et al., 2019). Darakhshan et al. revealed that the serum levels of angiostasis (CXCL9) are increased in GDM, while CXCL9 is decreased in neonates delivered by GDM(Darakhshan et al., 2019). Our study found a decrease in CXCL9 expression in the GDM group. It was found by You et al. (2025), after treating MIN6 cells with various combinations of IFN-γ, IL-1β, and TNFα, that CXCL9 production was not induced by any cytokine alone. Only combinations containing IFN-γ induced it, and the maximum production was achieved when all three cytokines were used together. Thus, the effect of CXCL9 on GDM needs to be further studied.

CXCL9 exerts its biological effects through binding to CXC chemokine receptor 3 (CXCR3), which is expressed on decidual NK cells, effector T lymphocytes, and extravillous trophoblasts at the maternal–fetal interface (Müller et al., 2010). CXCR3 engagement by CXCL9 activates downstream signaling cascades including JAK/STAT1/3, PI3K/Akt, and MAPK/ERK pathways (Müller et al., 2010), which collectively regulate chemotaxis, cell survival, and the transcriptional expression of additional immunomodulatory mediators. The downregulation of CXCL9 in GDM placentas would therefore impair CXCR3-dependent immune cell recruitment and positioning, potentially disrupting the coordinated spatial and functional organization of immune cells within the placental bed. Furthermore, attenuated CXCR3 signaling on trophoblast populations may compromise their immunomodulatory functions, contributing to the dysregulated maternal–fetal immune crosstalk characteristic of GDM. Direct measurement of CXCR3 expression and downstream phospho-signaling events in GDM placental tissues represents an important avenue for future investigation.

The IL1RL1 gene encodes ST2, a member of the IL-1 receptor superfamily. ST2 exists as a membrane-bound receptor (ST2L) that binds IL-33, and a soluble decoy receptor (sST2) that sequesters IL-33 (Griesenauer and Paczesny, 2017; Pascual-Figal and Januzzi, 2015). Mechanistically, IL-33 binding to ST2L recruits IL-1RAcP and initiates signaling through the MyD88/IRAK/TRAF6 axis, culminating in NF-κB and MAPK activation (Pinto et al., 2018). In the placental context, however, this pathway preferentially drives Th2-type cytokine production and ST2^+^ Treg expansion, rather than classical pro-inflammatory responses (Liew et al., 2016), thereby serving as a critical homeostatic mechanism for maintaining maternal–fetal immune tolerance. Downregulation of IL1RL1 in GDM would impair this regulatory circuit by reducing both ligand-responsive ST2L and the soluble decoy receptor sST2. The consequent loss of IL-33-mediated Treg expansion removes a major brake on Th1- and Th17-type effector responses, shifting the placental immune balance from a tolerance-dominant state to one permissive to inflammatory injury. This loss of counter-regulation is particularly consequential in GDM, where additional inflammatory drivers, including TLR4/NF-κB signaling and IL-1β-mediated inflammasome activation, are already operative, creating a self-amplifying cycle of placental inflammation and dysfunction.

In non-gestational models, IL-33 treatment has been shown to reduce immune cell infiltration and expand ST2-expressing Treg cells, thereby protecting against autoimmune diabetes (Pavlovic et al., 2018; Ryba-Stanisławowska et al., 2016; Lu et al., 2019; Zdravkovic et al., 2009). Extrapolating to pregnancy, the IL-33/ST2 pathway may play a key role in maintaining maternal–fetal immune tolerance and counteracting placental inflammation. In our validation cohort, IL1RL1 expression was significantly downregulated in GDM placentas. This reduction could impair IL-33/ST2-mediated regulatory signals, shifting the placental immune environment toward a pro-inflammatory state and contributing to GDM pathophysiology. However, direct functional evidence in human gestational tissues is lacking. Our findings identify IL1RL1 downregulation as a candidate signature of immune dysregulation in GDM, warranting further mechanistic studies using trophoblast cell lines or placental explants. Therefore, we speculate that CXCL9 and IL1RL1 are linked to the onset and progression of GDM and may play a role in its pathophysiology based on these results.

A noteworthy finding of this study is the simultaneous downregulation of CXCL9, a chemokine canonically associated with Th1-type responses, and IL1RL1, a receptor critical for Th2/Treg-mediated immune tolerance. At first glance, this bidirectional alteration appears paradoxical in the context of the pro-inflammatory milieu of GDM. We propose that this pattern reflects a broader state of immune regulatory dysfunction at the maternal–fetal interface, rather than unidirectional hyper-inflammation. The downregulation of IL1RL1 likely impairs IL-33/ST2-mediated tolerance signaling, removing a key counter-regulatory brake on inflammation. The concurrent downregulation of CXCL9 may signify a secondary deficit in IFN-γ-dependent immune coordination, compromising the placenta’s ability to appropriately position and activate immune effector cells. Together, these alterations may create a uniquely dysfunctional immune environment characterized by dysregulated inflammatory susceptibility and impaired host defense capacity — a concept consistent with the dual clinical features of heightened inflammation and increased infection risk observed in GDM pregnancies.

### Potential divergence between tissue and systemic expression of immune markers

We hypothesize that the downregulation of CXCL9 and IL1RL1 in GDM placentas, observed in our study, may reflect a localized failure of immunoregulatory signals at the maternal–fetal interface, rather than a uniform systemic inflammatory state. The previously reported elevation of serum CXCL9 in GDM patients could represent a distinct, potentially compensatory, systemic response. However, this tissue–systemic discrepancy remains speculative, and future studies measuring matched maternal blood, placental tissue, and cord blood are required to test this hypothesis directly.

In an underpowered exploratory blood dataset (GSE19649, 1 Non-GDM vs. 2 GDM), we observed a directional trend for IL1RL1 downregulation consistent with our placental findings, but no statistical conclusions can be drawn from this comparison (Supplementary Results, Supplementary Figure S2; Supplementary Table S8).

Finally, the CIBERSORT method was utilized by us to evaluate the types of infiltrating immune cells in the GDM. A significant increase in monocyte infiltration and a significant decrease in resting NK cells were detected, and GDM was potentially correlated with macrophage M1 infiltration. Direct or indirect contact between maternal immune cells and the placenta is brought about by the circulation of peripheral blood through the placenta. Distinct NK- and T-cell phenotypes altered in obese normal glucose tolerant (NGT) and non-obese GDM participants were simultaneously identified via unsupervised clustering of immune populations across the three biological sites, while a classical tissue monocyte cluster was significantly increased solely in obese GDM (Musumeci et al., 2024). It is demonstrated by Huang et al. that reduced monocyte count throughout pregnancy in women with GDM may contribute to GDM development, as insulin resistance is mediated via downregulation of anti-inflammatory factors, upregulation of inflammatory factors, and alteration of placenta-derived macrophage differentiation (Huang et al., 2022). Monocytes are widely recognized to migrate into tissues and differentiate into macrophages. GDM placentas with M1 macrophage phenotypes feature increased levels of IL-6, TNF-α, IL-1β, IL-8, and monocyte chemoattractant protein 1 (MCP-1), which significantly affect the inflammatory response (You et al., 2013; Mrizak et al., 2014). Our findings, along with previous evidence, indicate that CXCL9 and IL1RL1 genes—which are associated with altered immune cell infiltration—are significantly dysregulated in GDM and should be prioritized in future research.

Consistency across deconvolution methods strengthens immune dysregulation findings. Although CIBERSORT was initially validated for RNA-seq data, our complementary use of MCP-counter—a method optimized for microarray platforms—provides convergent evidence for altered immune landscapes in GDM placentas. The identification of reduced T lymphocyte infiltration by both methods supports the reliability of that observation. However, the discordant results for NK cells between CIBERSORT and MCP-counter highlight the importance of using multiple deconvolution algorithms and interpreting findings with caution. While absolute proportions may vary between algorithms, the directional agreement for key immune subsets supports the conclusion that GDM is associated with distinct shifts in placental immune cell composition, particularly involving innate and regulatory cell types.

### Discrepancy between discovery and validation datasets and lack of diagnostic utility

After correcting for pseudoreplication, the GSE70493 discovery dataset did not support the use of CXCL9 or IL1RL1 as diagnostic biomarkers. The AUC of CXCL9 was only 0.655 with a 95% confidence interval crossing 0.5, and its sensitivity and specificity were very low (20% and 40%, respectively). IL1RL1 was not significantly differentially expressed in this dataset (p = 0.21). These findings indicate that the discovery dataset is underpowered (n=30 GDM, n=25 Non-GDM) and the transcriptional effect sizes are small (as shown in our sensitivity analysis, Supplementary Figure S1), precluding reliable biomarker identification. In contrast, our independent validation cohort (n=50 per group) consistently demonstrated significant downregulation of both genes by qRT-PCR and IHC. This discrepancy may be explained by the larger sample size and higher sensitivity of the validation methods, as well as possible batch effects or placental sampling heterogeneity. Therefore, while the discovery dataset does not support diagnostic claims, the external validation provides evidence that CXCL9 and IL1RL1 are biologically relevant to GDM-associated immune dysregulation. Future studies should employ well-powered, multi-center cohorts to further evaluate their potential as adjunctive markers or therapeutic targets.

Several limitations should be acknowledged. First, the discovery dataset (GSE70493) had limited statistical power (n=30 GDM, n=25 Non-GDM) after correction for technical replicates, and no gene survived FDR correction, which likely explains the non-significant IL1RL1 result in this dataset despite its consistent downregulation in our larger validation cohort. Second, although the validation cohort (n=50 per group) supports the downregulation of CXCL9 and IL1RL1 in GDM placentas, larger multi-center studies are needed for confirmation. Third, our validation was tissue-based; circulating levels of these markers remain to be assessed in adequately powered blood-based studies. Fourth, the discovery dataset did not support any diagnostic utility for CXCL9 or IL1RL1 (AUC=0.655, 95% CI crossing 0.5; IL1RL1 non-significant). Fifth, although we increased the qRT-PCR sample size to 38 (19 GDM, 19 Non-GDM), this remains moderate and larger fresh-tissue cohorts are warranted. Sixth, our discovery relied on a single GEO dataset (GSE70493). Other placental datasets (GSE128381, GSE154377, GSE9984, GSE103552) were unsuitable for independent validation due to missing GDM annotation, non-placental origin, or small sample sizes. Therefore, computational generalizability beyond GSE70493 requires future multi-center studies. However, our independent qRT-PCR and IHC validation (50 GDM/50 Non-GDM) supports the biological findings. Moreover, the functional mechanisms linking CXCL9 and IL1RL1 downregulation to GDM pathogenesis require further investigation using trophoblast cell lines, placental explants, or animal models. Additionally, although GAPDH has been widely used and validated as a reference gene in prior GDM placental studies, future investigations would benefit from systematic screening of multiple candidate reference genes using stability analysis algorithms such as geNorm or NormFinder to further confirm normalization accuracy. Furthermore, as pre-pregnancy BMI differed significantly between the GDM and control groups, and given that obesity itself can influence placental immune homeostasis, we cannot fully exclude the possibility that BMI partially contributed to the observed gene expression changes. Future studies with larger sample sizes should incorporate multivariable adjustment for BMI and other metabolic confounders to isolate the effects attributable to GDM pathology. Finally, while women with clinical chorioamnionitis, intrahepatic cholestasis of pregnancy, and documented active infections were excluded, we did not systematically screen for subclinical infections, which may represent an unmeasured confounder of the placental immune profile.

## Conclusion

This study identifies CXCL9 as a consistently downregulated immune-related gene in GDM placentas, with a small-to-moderate effect size (Cohen’s d = −0.718) but no diagnostic utility in the discovery dataset. IL1RL1 showed discordant results: non-significant in the underpowered discovery cohort yet significantly downregulated in a larger independent validation cohort, highlighting the importance of adequate sample size and external validation in transcriptomic biomarker studies. Neither gene met the criteria for clinical diagnostic application. Instead, these findings support an association of CXCL9 and IL1RL1 with GDM-related immune dysregulation. Larger, multi-center, and mechanism-driven studies are required to determine whether these molecules represent therapeutic targets or adjunctive markers in GDM.

## Data Availability

The original contributions presented in the study are included in the article/Supplementary Material, further inquiries can be directed to the corresponding authors.
