# Supplementary figures and images for: Immune dysregulation in gestational diabetes mellitus: placental downregulation of CXCL9 and IL1RL1 and altered immune cell infiltration
Source: Front Cell Dev Biol. 2026 May 29;14:1803128. doi: 10.3389/fcell.2026.1803128 (PMC13260586; doi:10.3389/fcell.2026.1803128)

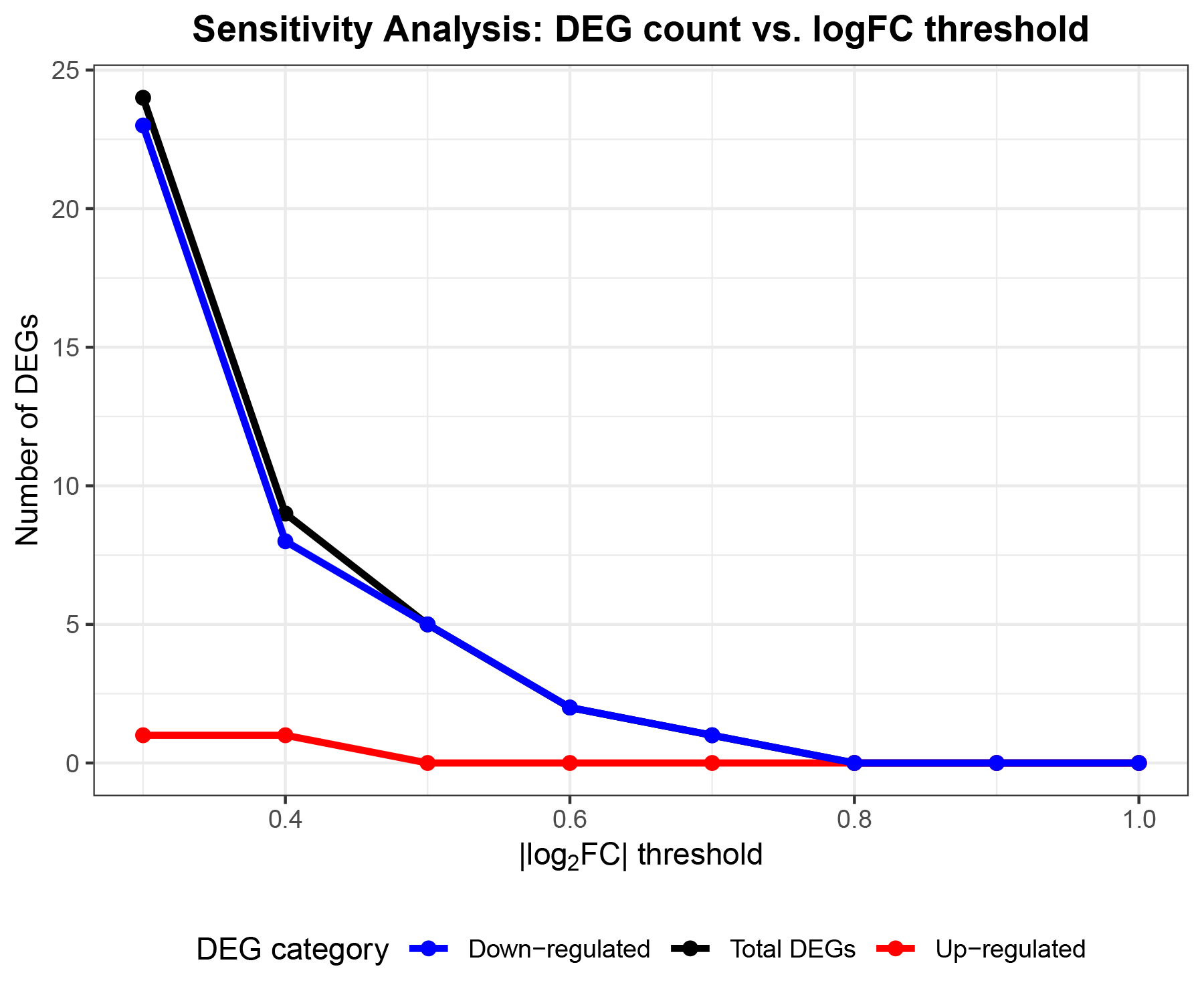

Supplement: Supplementary file 1 [file DataSheet1.zip › supplementary materials/Figure S1 Sensitivity analysis of DEGs.tif]

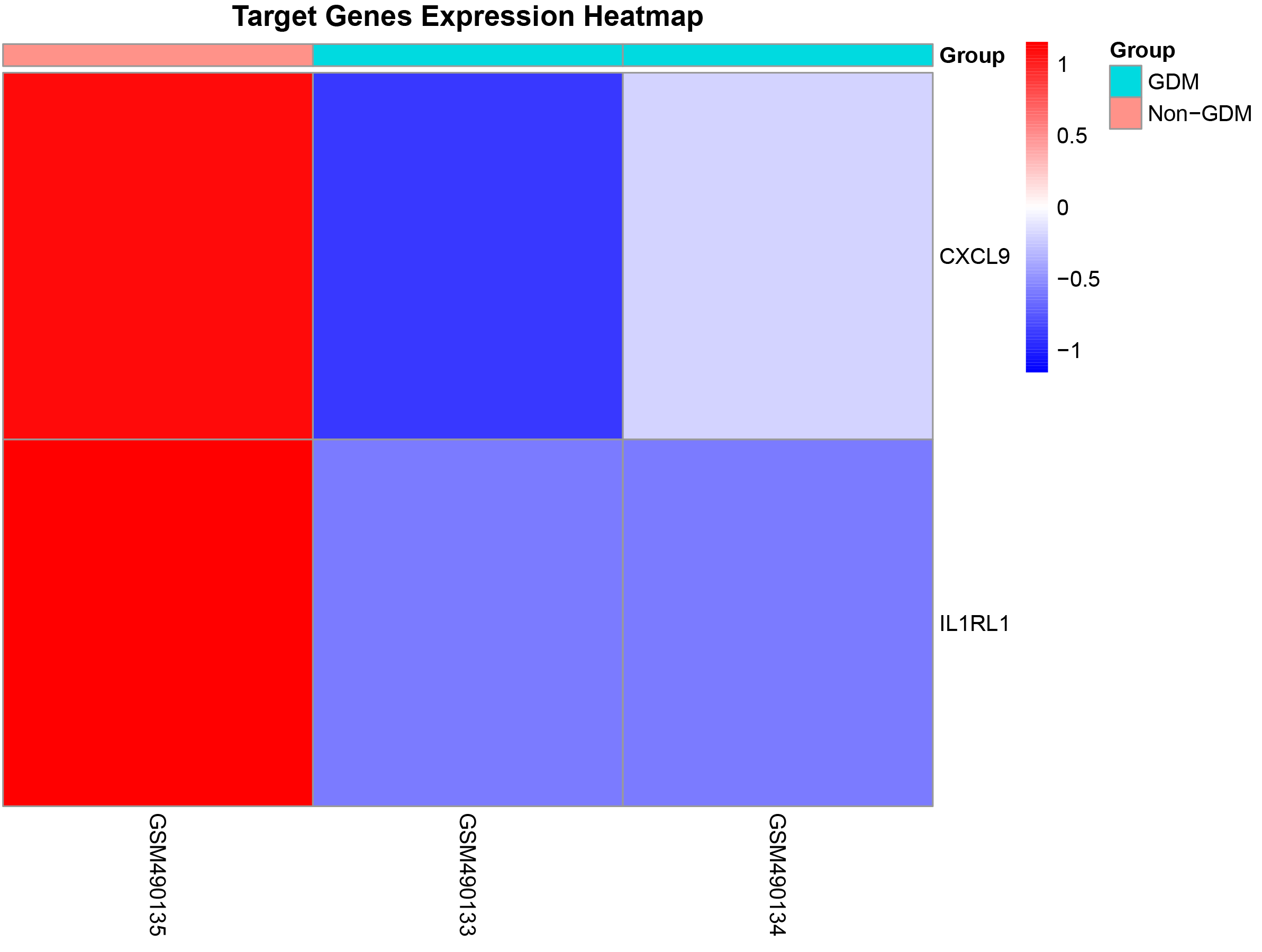

Supplement: Supplementary file 1 [file DataSheet1.zip › supplementary materials/Figure S2 Expression heatmapof CXCL9 and IL1RL1 genes.tif]
