# Supplementary material for: Immune dysregulation in gestational diabetes mellitus: placental downregulation of CXCL9 and IL1RL1 and altered immune cell infiltration
Source: Front Cell Dev Biol. 2026 May 29;14:1803128. doi: 10.3389/fcell.2026.1803128 (PMC13260586; doi:10.3389/fcell.2026.1803128)
Supplement: Supplementary file 1 [file DataSheet1.zip › supplementary materials/supplementary result.doc]

Supplementary Results (new subsection):

Exploratory observation in a whole‑blood dataset (GSE19649). To preliminarily explore whether the downregulation of CXCL9 and IL1RL1 observed in placental tissue might also be detectable in the systemic circulation, we examined their expression in the publicly available whole‑blood dataset GSE19649. This dataset contains only 3 samples in total (1 normoglycemic control, 2 GDM). As an exploratory observation with no inferential power, we noted a directional trend for IL1RL1 downregulation in GDM (log_2_FC = -1.49) and a similar but weaker trend for CXCL9 (log_2_FC = -0.38) (Figure S2, Table S8). However, no statistical conclusions can be drawn from this comparison due to the extremely limited sample size. These descriptive observations are presented solely to generate hypotheses for future adequately powered blood-based studies.
